# Regulation and Role of GLI1 in Cutaneous Squamous Cell Carcinoma Pathogenesis

**DOI:** 10.3389/fgene.2019.01185

**Published:** 2019-12-04

**Authors:** Joanna Pyczek, Natalia Khizanishvili, Maria Kuzyakova, Sebastian Zabel, Julia Bauer, Frauke Nitzki, Steffen Emmert, Michael P. Schön, Petra Boukamp, Hans-Ulrich Schildhaus, Anja Uhmann, Heidi Hahn

**Affiliations:** ^1^Institute of Human Genetics, University Medical Center Goettingen, Goettingen, Germany; ^2^Department of Dermatology and Venerology, University Medical Center Rostock, Rostock, Germany; ^3^Department of Dermatology, Venerology and Allergology, University Medical Center Goettingen, Goettingen, Germany; ^4^Division of Genetics of Skin Carcinogenesis, German Cancer Research Center (DKFZ) Heidelberg, Germany; ^5^Institute for Pathology, University Medical Center Goettingen, Goettingen, Germany

**Keywords:** Hedgehog signaling, cutaneous squamous cell carcinoma, GLI1, epidermal growth factor receptor, ERK

## Abstract

Cutaneous squamous cell carcinoma (cSCC) is the second most common skin tumor in humans. Although current therapies are sufficient to clear the tumor in many cases, the overall risk of cSCC metastasis is still 5%. Alternative treatment options could help to overcome this situation. Here we focused on the role of the Hedgehog (HH) signaling pathway and its interplay with epidermal growth factor receptor (EGFR) signaling in cSCC. The analyses revealed that, despite lack of Sonic HH (SHH) expression, a subset of human cSCC can express *GLI1*, a marker for active HH signaling, within distinct tumor areas. In contrast, all tumors strongly express EGFR and the hair follicle stem cell marker SOX9 at the highly proliferative tumor-stroma interface, whereas central tumor regions with a more differentiated stratum spinosum cell type lack both EGFR and SOX9 expression. *In vitro* experiments indicate that activation of EGFR signaling in the human cSCC cell lines SCL-1, MET-1, and MET-4 leads to *GLI1* inhibition *via* the MEK/ERK axis without affecting cellular proliferation. Of note, EGFR activation also inhibits cellular migration of SCL-1 and MET-4 cells. Because proliferation and migration of the cells is also not altered by a *GLI1* knockdown, GLI1 is apparently not involved in processes of aggressiveness in established cSCC tumors. In contrast, our data rather suggest a negative correlation between *Gli1* expression level and cSCC formation because skin of *Ptch* *^+/-^* mice with slightly elevated *Gli1* expression levels is significantly less susceptible to chemically-induced cSCC formation compared to murine wildtype skin. Although not yet formally validated, these data open the possibility that GLI1 (and thus HH signaling) may antagonize cSCC initiation and is not involved in cSCC aggressiveness, at least in a subset of cSCC.

## Introduction

Cutaneous squamous cell carcinoma (cSCC) is the second most common human skin tumor after basal cell carcinoma (BCC) with increasing incidence. In Europe, the highest cSCC incidences are recorded in the United Kingdom and the Netherlands with approximately 32 and 22–35/100.000 new cases per year (Leiter et al., 2016). Although there are efficient methods of treatment, approximately 8% of cSCC will reappear. In addition, approximately 5% of these tumors will metastasize (Leiter et al., 2016) and of these patients, 40% will die (Voiculescu et al., 2016). The increasing cSCC incidence and the risk of recurrence or metastasis show that a better understanding of the molecular basis of this disease is important to improve current treatments.

cSCC are characterized by malignant proliferation of epidermal keratinocytes. They are heterogeneous invasive tumors that show proliferation at the tumor-stroma interface and usually have an inner differentiating cell mass resembling the stratum spinosum. Usually cSCC are induced by ultraviolet radiation and emerge from *in situ* lesions such as actinic keratosis or Bowen’s disease. Like in BCC, the potential cellular origins of cSCC include the SOX9-positive hair stem cell compartment encompassing the bulge region of the hair follicle and the basal layer of the interfollicular epidermis (Vidal et al., 2005; Ratushny et al., 2012). Indeed, cSCC express SOX9, which induces proliferation of keratinocytes (Shi et al., 2013), deregulates hair follicle stem cell maintenance and suppresses epidermal differentiation (Kadaja et al., 2014). Furthermore, 43% of locally-advanced and 80–100% of metastatic cSCC express epidermal growth factor receptor (EGFR) (Shimizu et al., 2001; Maubec et al., 2005; Fogarty et al., 2007a). EGFR expression is also associated with lymph node metastasis and progression and thus has prognostic implications in cSCC (Canueto et al., 2017). The two main pathways activated by EGFR signaling are the RAS/RAF/MEK/ERK cascade and the PI3K/AKT axis, which are involved in proliferation, differentiation, apoptotic processes and cell metabolism (reviewed in Shaul and Seger, 2007; Manning and Toker, 2017). Indeed, cSCC show phosphorylation of the EGFR-downstream signaling targets ERK (Rittie et al., 2007; Zhang et al., 2007; Sonavane et al., 2012), AKT (Rittie et al., 2007; Barrette et al., 2014), and S6 (Khandelwal et al., 2016). Based on these data, EGFR itself and its downstream signaling pathways seem to be a promising target for cSCC therapy. Consequently, the EGFR-directed monoclonal antibody cetuximab is currently applied in clinical trials (Dereure et al., 2016; Wollina et al., 2018).

Recently, the HH signaling pathway has been implicated in cSCC pathology. HH signaling not only plays an important role in skin development but also in skin cancer. Thus, inactivating mutations in the HH receptor and tumor suppressor gene *Patched1* (*PTCH*) are found in the majority of sporadic BCC cases, where they function as driver mutations (for review see Epstein, 2008). *PTCH* mutations have also been identified in some cases of cSCC (Ping et al., 2001). Furthermore, cSCC have been reported to express major components/proteins of the HH pathway including Sonic Hedgehog (SHH), PTCH, and the major target of active HH signaling GLI1 (Schneider et al., 2011; Tanese et al., 2018). On the other hand, cSCC mouse models suggest that Ptch paradoxically can act as an oncogene in cSCC and promotes the formation of cSCC (Wakabayashi et al., 2007; Kang et al., 2013). Thus, the role of HH signaling in cSCC is far from understood.

Canonical HH signaling comprises binding of HH to the PTCH receptor, activation and accumulation of the transmembrane protein Smoothened (SMO) at the primary cilium and translocation of the GLI2/GLI3 transcription factors into the nucleus. One of the major targets of the HH pathway is GLI1, which amplifies the HH signal in a positive feedback (for review see e.g. Aberger et al., 2012; Pandolfi and Stecca, 2015). Activation of HH signaling can also occur non-canonically in that GLI activity is regulated independently of PTCH and SMO. Non-canonical activation of HH signaling can be triggered by growth factors and their downstream signaling axes RAS/RAF/MEK/ERK and PI3K/AKT/mTOR. However, these factors can also inhibit the HH pathway, which apparently depends on the cellular context. Examples are oncogenic *KRAS* mutations, which tumor-intrinsically inhibit HH signaling but simultaneously activate it in the tumor microenvironment (Lauth et al., 2010). Other examples are fibroblast growth factor (FGF) and EGFR signaling. Whereas FGF counteracts HH/GLI-dependent proliferation and growth of medulloblastoma (Fogarty et al., 2007b; Emmenegger et al., 2013), EGF is essential in determination of the oncogenic phenotype of HH/GLI-driven BCC (Schnidar et al., 2009; Eberl et al., 2012). However, the role of EGFR signaling might be different in cSCC, because EGF has been shown to inhibit growth of cSCC cell lines (Barnes, 1982; Gill et al., 1982; Ponec et al., 1988; Commandeur et al., 2012).

Here we thoroughly reexamined the role of HH signaling in cSCC by using *GLI1* as a read-out and analyzed it’s interaction with EGFR. Human cSCC samples highly express EGFR, whereas *GLI1* is only expressed in distinct tumor areas. Indeed, in our cell culture settings EGFR signaling blocks *GLI1* expression *via* the MEK/ERK axis. This does not affect cellular proliferation but can inhibit cSCC cell migration in a GLI1-independent manner, suggesting that GLI1 is not involved in cSCC progression. Interestingly, elevated Gli1 levels in the skin rather are associated with prevention of cSCC initiation, at least when these tumors are induced chemically in the mouse.

## Materials and Methods

### Biopsies

Ten biopsy specimens from invasive cSCC were studied. Histopathology of all cases was centrally reviewed. Materials were obtained at a time when patients gave their general consent to use of their materials for scientific purposes, so additional informed consent for this particular study was required. The respective approval by our local ethics committee has been obtained for staining of tumor markers in epithelial skin tumors. The study was approved by the ethics and review committee of the University Goettingen (file number 19/3/02).

### Immunohistochemistry and *In* *Situ* Hybridization

Formalin-fixed human cSCC samples (n = 10) were embedded in paraffin and sectioned at 5 µm for histological analyses. Hematoxylin and eosin staining was performed according to standard methods. Antigen retrieval methods, antibodies and antibody dilutions used for immunohistochemical stainings are listed in **Supplementary Table S1**. Immunofluorescent stainings of cells with acetylated α-tubulin to detect primary cilia was done as described previously (Geyer et al., 2018). For *GLI1 in situ* hybridization, the RNAscope^®^ technology was performed using the RNAscope^®^ Probe Hs-GLI1 (310991) and the RNAscope^®^ 2.5 HD Reagent Kit-RED (322350; Advanced Cell Diagnostics) according to the manufacturer’s recommendations. The RNAscope^®^ Probes Hs-PPIB (313901) and DapB (310043) served as positive and negative controls, respectively.

### Real-Time Quantitative Polymerase Chain Reaction

Total RNA from murine back and tail skin or from cell lines was extracted using RNA fibrous tissue kit (Qiagen) or TRIzol reagent (Thermo Fisher Scientific), respectively. Complementary DNA was synthesized using Superscript II and random hexamers (Invitrogen). Gene expression was quantified by SYBR Green-based real-time quantitative polymerase chain reaction (qRT-PCR) assays by the ABI Prism HT 7900 Detection System instrument and software (Applied Biosystems). Data were analyzed by the standard curve method for relative quantification. The primers for amplification of target transcripts are described in **Supplementary Table S2**. Primer pairs were intron-flanking, except for the primers for *18S* ribosomal RNA (rRNA) and *SHH* transcripts. Amplification of *18S* rRNA messenger RNA (mRNA) served to normalize the amount of sample complementary DNA.

### Cell Culture Experiments

The human cSCC cell lines SCL-1, SCL-2, SCC-12, SCC-13, MET-1, and MET-4 were cultured in Roswell Park Memorial Institute medium, 10% fetal calf serum (FCS), 1% non-essential amino acids, 200 nM L-glutamine, and 1% penicillin/streptomycin (PS) (SCL-1, SCL-2, and SCC-13) or in Dulbecco’s modified Eagle medium (DMEM), 10% FCS, and 1% PS (SCC-12, MET-1, and MET-4). Shh-responsive *Ptch* *^flox/flox^* *ERT2* *^+/-^* fibroblasts, NIH/3T3, DAOY, and HT1080 cells were maintained in DMEM, 10% FCS, and 1% PS. RMS13 cells were maintained in Roswell Park Memorial Institute medium, 10% FCS, and 1% PS.

Hedgehog Antag (HhA) and GDC-0941 were from Genentech (San Francisco, CA, USA), vismodegib (GDC-0449), SCH772984, and MK-2206 from Selleckchem (Munich, Germany), cyclopamine, GANT61, and everolimus from Sigma-Aldrich (Steinheim, Germany), rapamycin from Calbiochem, Merck KGaA (Darmstadt, Germany), PI103 from Axxora Deutschland GmbH (Lörrach, Germany), UO126 from Cell signaling (Danvers, MA, USA), SAG from Cayman chemicals (Ann Arbor, MI, USA), and EGF was from R&D System (Minneapolis, MN, USA). For *in vitro* assays the drugs were dissolved in dimethyl sulfoxide (DMSO) (HhA, vismodegib, GANT61, rapamycin, GDC-0941, PI103, MK-2206, UO126, SCH772984, and SAG), in ethanol (cyclopamine and everolimus) or in 0.1% bovine serum albumin/10 mM acetic acid (EGF). All compounds were easy to solubilize in the respective solvents. The final DMSO and ethanol concentrations were equal in all experimental settings i.e. 0.1% DMSO and 0.1% ethanol. The final drug concentrations used for *in vitro* analysis are indicated in the respective experiments.

For generation of Shh conditioned medium (Shh-CM) and respective control medium (control-CM), stably transfected HEK293-Shh (Chen et al., 2002) and non-transfected HEK293 cells were incubated in serum-depleted medium (DMEM supplemented with 2% FCS and 1% P/S). After 24 h the medium was collected and filtered through a 0.2 µm pore size disposable filters. The medium was stored at 4°C for a maximum of 4 months and tested prior use on Shh-responsive *Ptch* *^flox/flox^* *ERT2* *^+/-^* cells (see **Figure 2B** and Uhmann et al., 2011).

For all experiments using EGF, cells were grown for at least 24 h in starvation medium containing 0.5% FCS. Then EGF was added for the time frames as indicated in the respective experiments.

For gene expression analysis 2 × 10^5^ cells/well were seeded in 6-well-plates. After 24 h, the cells were washed, treated with drugs as indicated in the respective experiments for 24 h and harvested in TRIzol reagent for subsequent isolation of mRNA. For combined treatment with the inhibitors and EGF, the cells were incubated with the indicated inhibitors for the total time of 24 h and EGF was added for the last 3 h of incubation.

For WST assay 6 × 10^3^ cells/well were seeded in 96-well-plates. After 24 h, the cells were washed, treated with respective drugs for the total time of 24 h and incubated with WST-1 (1:25 in respective cell culture medium) for the last 3 h of treatment. The intensity of the signal was measured in a microplate reader at a wavelength of 450 and 655 nm (background signal).

For bromodeoxyuridine (BrdU) incorporation assays 6 × 10^3^ cells/well were seeded into 96-well-plates. After attachment of the cells for 24 h, the cells were incubated for 24 h with medium supplemented with the indicated inhibitors and 10 µM BrdU. Cellular proliferation after BrdU-pulsing was measured using a Cell Proliferation BrdU ELISA (Roche Diagnostics GmbH). BrdU-incorporation is presented as the percentage of the incorporation measured in time-matched vehicle-treated control cells (that was set to 100%).

For dual luciferase assay 3 × 10^4^ cells/well were seeded in 24-well-plates. After 24 h cells were transfected (see below) with *p9xGli-BS* encoding firefly luciferase under the HSV TK promoter containing 9 Gli-binding sites. A *Renilla* reporter plasmid was used for normalization. After transfection, the cells were incubated with Shh-CM or control-CM and harvested 24 h later when the cells were confluent. Co-transfection with *pGli1* served as positive control as described previously (Pyczek et al., 2016). Luciferase activity was measured in 96-well plate using the Dual Luciferase Assay Kit (Promega) according to the manufacturer’s protocol.

For analyses of HH signaling activity and BrdU incorporation after *GLI1* knock down or *Gli1*/*GLI1* overexpression, SCL-1 and/or MET-4 cells were transfected with *GLI1*-specific small interfering RNA (siRNA) (Dharmacon ON-TARGETplus SMARTpool), scrambled siRNA (AllStars negative, Qiagen) (300 ng siRNA/1.5 × 10^5^ cells), plasmids encoding murine or human *Gli1/GLI1* or/and plasmids for dual luciferase assay (4 µg DNA/1.5 × 10^5^ cells) using the transfection reagent HiPerfect (Qiagen) for siRNA transfection or RotiFect (Carl Roth) for plasmid DNA transfection according to the manufacturer’s instructions. MET-4 were incubated for 6 h, SCL-1 for 12 h with the respective transfection media/RNA or DNA mixture before medium was replaced by the respective culture medium. 24 h thereafter the cells were harvested for gene expression analysis or re-seeded for BrdU incorporation assay.

Cellular migration after transfection with *GLI1*-specific siRNA or after treatment with EGF was measured by the zone exclusion assay Oris^™^ Cell Migration assay kit (Platypus technologies) and a SynergyMx (BioTek) plate reader according to the manufacturer’s instructions. For this purpose trypsinized cells were labeled with 5 µM CellTracker^™^ Green CMFDA Dye (Invitrogen) in DMEM without supplements at 37°C, and in a humidified atmosphere with 5% CO_2_ for 30 min. For EGF treatment experiments 3 × 10^4^ SCL-1, 4 × 10^4^ MET-1 or MET-4 CellTracker^™^-labeled cells/well were seeded in the respective media supplemented with 0.5% FCS in 96-well-Oris^™^ Cell Migration assay kit plates. After a 24 h starving period the stoppers were removed, cells were washed in 1 × PBS and maintained for additionally 18 h in starving medium supplemented with 100 ng/µl EGF or solvent. Cellular migration was quantified in paraformaldehyde-fixed cells by measuring the fluorescence intensity within the detection zone using the detection mask. As controls 3 × 10^4^ HT1080 CellTracker^™^-labeled cells/well were seeded in DMEM supplemented with 10% FCS for 24 h. After stopper removal, HT1080 cells were maintained for additionally 12 h in 0.5% or 10% FCS until cellular migration was measured. Reference wells, in which the stoppers were retained until results were read, served as pre-migration controls in each experiment.

For *GLI1* knock down experiments 1 × 10^6^ CellTracker^™^-labeled MET-4 were transfected ahead of seeding with 300 ng *GLI1*-specific (Dharmacon, ON-TARGETplus SMARTpool) or scrambled siRNA (Dharmacon, ON-TARGETplus Non-targeting Pool) using the NeonTransfection System (Invitrogen) according to the manufacturer’s instructions and the following transfection conditions: 1 pulse at 1,400 V and a pulse width of 30 ms. Afterwards 80,000 CellTracker^™^-labeled siRNA-transfected MET-4 cells/well were seeded in the respective medium supplemented with 0.5% FCS in 96-well-Oris^™^ Cell Migration assay kit plates. After 24 h the stoppers were removed, cells were washed in 1 × PBS and maintained for additional 18 h in starving medium and cellular migration was analyzed as described above. For verification of the siRNA-mediated *GLI1* knock down 360,000 of the remaining CellTracker^™^-labeled siRNA-transfected MET-4 cells were seeded in 1 well of a 6-well plate in starving medium. RNA was harvested at the same time point as the cellular proliferation was measured.

The data shown summarize three independent experiments performed at least in triplicates. Graphs represent the mean value of all measurements + SEM.

### Western Blot Analysis

Preparation of cell lysates and determination of protein concentrations were done as described previously (Marklein et al., 2012). Primary antibodies used to detect the individual target proteins and corresponding secondary antibodies are given in **Supplementary Table S1**. All Western blots shown are representative for at least two independent experiments.

To separate the nuclear and cytoplasmic fraction, cells were resuspended in 250 µl of subcellular fractionation buffer [250 mM sucrose, 20 mM 4-(2-hydroxyethyl)-1-piperazineethanesulfonic acid at pH 7.4, 10 mM potassium chloride, 1.5 mM magnesium chloride, 1 mM ethylenediaminetetraacetic acid, 1 mM egtazic acid, 1 mM dithiothreitol, phosphatase, and protease inhibitor] and passed several times through a 30G needle using a 1 ml syringe. Lysis was performed 45 min on ice. After centrifugation at 3,000 rpm and 4°C for 15 min, the respective supernatant was again centrifuged at 8,000 rpm and 4°C for 12 min and then contained the cytosolic and membrane fractions. The respective pellets were washed at 3,000 rpm and 4°C for 15 min in subcellular fractionation buffer, snap frozen in liquid nitrogen, thawed on ice and resuspended in 50 µl of nuclear lysis buffer (50 mM Tris-hydrochloride at pH 8, 50 mM Sodium chloride, 1% NP-40, 0.5 M sodium deoxycholate, 0.1% sodium dodecyl sulfate, 10% glycerol). After 45 min incubation on ice, the samples were centrifuged again at 3000 rpm and 4°C for 30 min and the supernatants containing the nuclear proteins were collected.

### Mice

Wildtype C57BL/6N and heterozygous *Ptch* mutant (*Ptch* *^+/-^*) mice on a pure C57BL/6N background were used. Genotyping of the mice was conducted by PCR on genomic DNA isolated from tail biopsies as previously reported (Zibat et al., 2009).

7, 12 - Dimethylbenz [a] anthracene (DMBA) / 12 - O - tetradecanoylphorbol - 13-acetate (TPA) treatment of heterozygous *Ptch* *^+/-^* (n = 5) and *Ptch* *^+/+^* (n = 8) control mice was conducted as recently described (Uhmann et al., 2014). In short, the flanks of 8-weeks old mice were shaved 2 days prior to a single topical application of 200 nM DMBA (dissolved in 200 µl acetone) and then once a week during the whole treatment time. One week after DMBA application, 20 nM TPA (in 50 µl acetone) was applied twice a week for 26 weeks. The tumor number per mouse was recorded twice weekly. After the last TPA treatment the animals were sacrificed and the treated skin areas were preserved for histology. DMBA/TPA treatments of *Ptch* mice were performed in compliance with all German legal and ethical requirements and have been approved by the Lower Saxony State Office for Consumer Protection and Food Safety (file number 33.9-42502-04-100/07).

### Statistical Analysis

If not indicated otherwise, statistical differences were analyzed using the software GraphPad Prism 6 by nonparametric Mann–Whitney testing. Data was considered significant when *P* < 0.05.

## Results

### Low *GLI1* Expression in Discrete Cutaneous Squamous Cell Carcinoma Tumor Nests and High EGFR, pS6, Ki67, and SOX9 Expression At the Tumor–Stromal Interface

**Figure 1:**
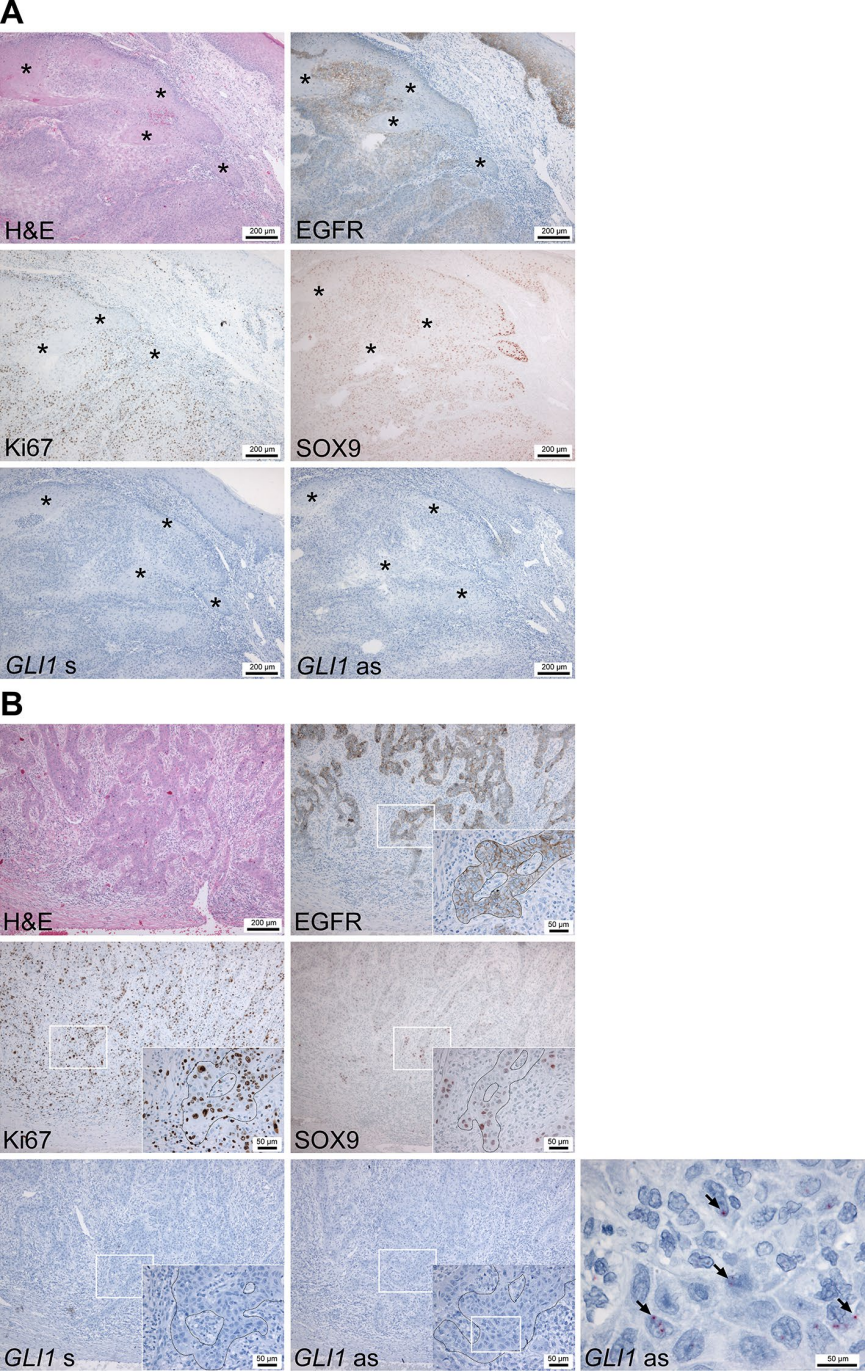
*GLI1* expression analyses of human cSCC biopsies. **(A** and **B)** Representative adjacent sections of two human cSCC biopsies stained with hematoxylin/eosin, antibodies against EFGR, Ki67 or SOX9 or sense (*GLI1* s) or antisense (*GLI1* as) RNA-probes against the *GLI1* transcript. All antibody stainings were visualized using AEC reagent (red), *GLI1* mRNA expression was detected by RNAscope^®^ *in situ* hybridization (red). Nuclei of sections analyzed *via* immunohistology and *in situ* hybridization were counterstained with hemalaun (blue). Specificity of the *GLI1* RNAscope^®^ *in situ* hybridization was verified by simultaneous analyzed BCC biopsies (see **Supplementary Figure S1B**) and by *GLI1* *^+^* hair follicles within the analyzed cSCC samples (see **Supplementary Figure S1C**). Asterisks in **(A)** mark differentiated and keratinized tumor areas. Arrows in **(B)** indicate *GLI1* *^+^* cSCC cells. White boxes in **(B)** indicate the magnification area shown in the insets or in the right panel.

To gain insight into the role of HH signaling in cSCC, immunohistochemical analyses of adjacent sections of 10 human invasive cSCC samples were performed. Initial anti-SHH antibody staining revealed that cSCC tumor cells as well as stromal cells were SHH negative (data not shown) although the malignant epithelium of human pancreatic adenocarcinoma sections showed strong positivity and thus resembles the described SHH expression pattern (Thayer et al., 2003) (**Supplementary Figure S1A**). We also stained the sections with an anti-IHH and two anti-DHH antibodies (**Supplementary Table S1**), which however either gave highly unreliable results or did not work at all (data not shown). However, when we measured the HH signaling activity in the cSCC samples *GLI1* specific *in situ* hybridization using the RNAscope^®^ technology (for positive controls see **Supplementary Figures S1B, C**), we found *GLI1* expression in discrete tumor nests in approximately 50% of tumors with a rather heterogeneous pattern (**Figures 1A, B**, lower right panels). Immunohistological antibody staining using anti-GLI1 C68H3 antibody from Cell Signaling, which has been described to work on skin tumor samples (Tanese et al., 2018), show that cSCC are either completely positive (3/10) or negative (4/10) for GLI1, whereas others show both GLI1-positive and GLI1-negative tumor areas (3/10) (**Supplementary Figure S2C**). Again, these data should be interpreted with great caution because anti-GLI1 antibody stainings of normal human skin and human BCC did not match with data from the literature (described in detail in **Supplementary Figures S2A, B**). In contrast, all tumors, and especially cells at the tumor–stroma interface, stained strongly positive for EGFR (**Figures 1A, B**, upper right panels) and its downstream target pS6 (**Supplementary Figure S1D**). Moreover, cells at the invasive tumor-stroma interface were also positive for Ki67 and for the inhibitor of epidermal differentiation SOX9 (Kadaja et al., 2014) (**Figures 1A, B**, middle panels) whereas keratinized or well differentiated tumor cells were rarely positive for these markers (**Figure 1A**, asterisks). Finally, all cSCC samples showed high EGF expression without any specific distribution (**Supplementary Figure S1E**).

Under the assumption that *GLI1* mRNA expression is the most reliable indicator of the HH pathway’s activity (for review see Hooper and Scott, 2005; Scales and de Sauvage, 2009), these data suggest that HH signaling is not prominent in cSCC, whereas EGF/EGFR signaling seems to be associated with a more aggressive and dedifferentiated cSCC phenotype.

### Canonical Hedgehog Signaling Cannot Be Activated in Cutaneous Squamous Cell Carcinoma Cell Lines

**Figure 2:**
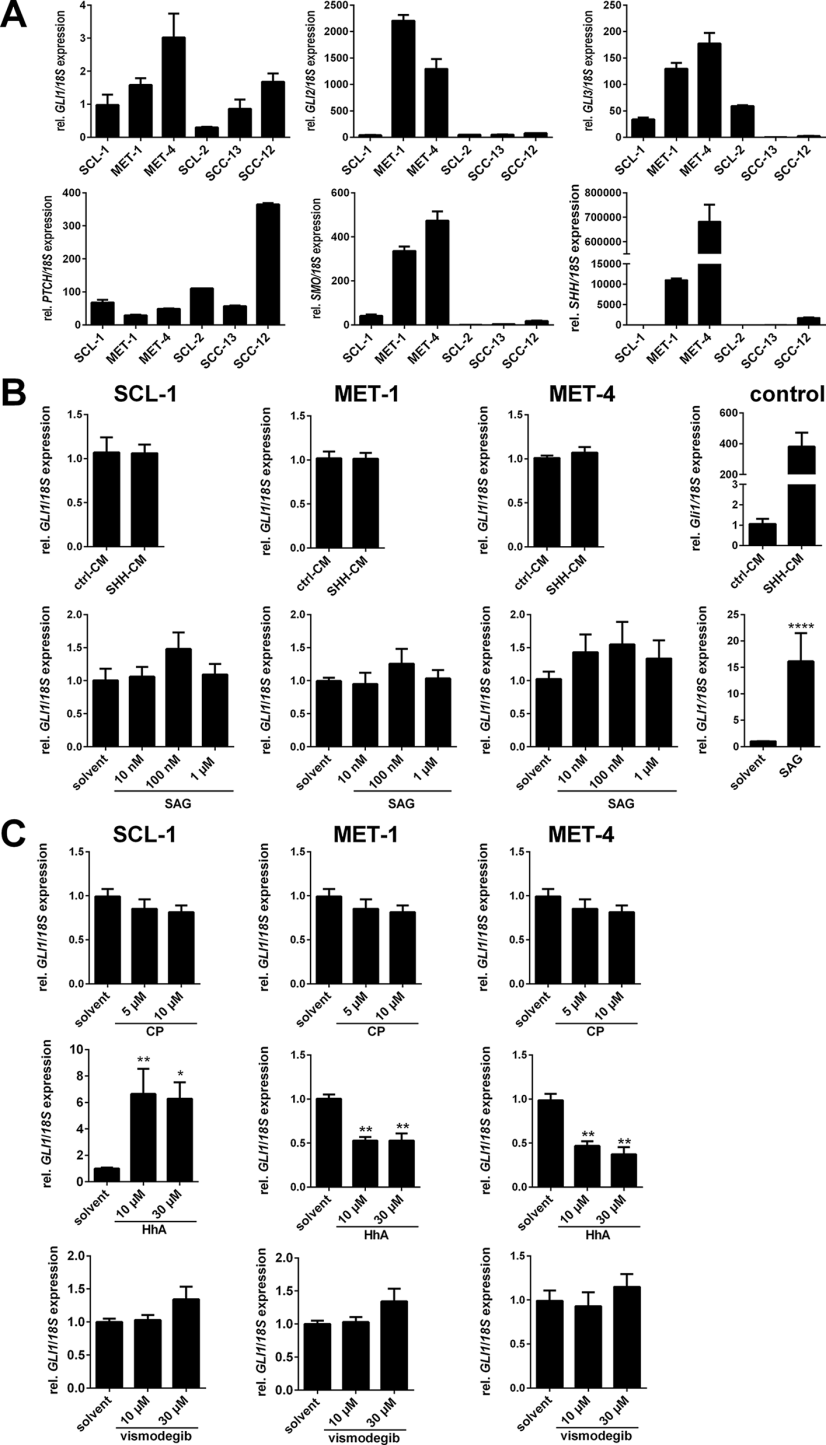
Impact of HH signaling activation or inhibition on human cSCC cell lines. **(A)** qRT-PCR-based *GLI1*, *GLI2*, *GLI3*, *PTCH*, *SMO*, and *SHH* expression analyses of the human cSCC cell lines SCL-1, MET-1, and MET-4. Gene expression levels were normalized to *18S rRNA* gene expression. Results represent mean values + SEM of one experiment measured in triplicates. **(B** and **C)** qRT-PCR-based *GLI1* expression analyses of SCL-1, MET-1, and MET-4 cells after 24 h incubation with **(B)** HH signaling activating molecules: control- (ctrl-CM) or Shh-conditioned medium (Shh-CM; upper panel) or Sonic Hedgehog agonist (SAG; lower panel) or with **(C)** HH signaling inhibiting molecules: cyclopamine (CP; upper panel), HhAntag (HhA; middle panel) or vismodegib (lower panel) at the indicated concentrations. The functionality of the HH signaling activating molecules was verified by Shh-CM-treatment of the Shh-responsive murine cell line *Ptch* *^flox/flox^* *ERT2* *^+/-^* [**(B)**, upper right panel] (Uhmann et al., 2011) and SAG-treatment of the SAG-responsive medulloblastoma cell line Daoy [**(B)**, lower right panel] (Gotschel et al., 2013). *GLI1* expression levels were normalized to *18S *rRNA gene expression and values of solvent treated controls were set to 1. Results represent mean values + SEM of two **(B)** or three **(C)** independent experiments measured in triplicates. Statistical significance was tested by a nonparametric Mann-Whitney test. *P < 0.05, **P < 0.01, ****P < 0.0001.

Since the *GLI1* expression seems to play only a minor role in cSCC we also investigated the mRNA expression level of HH signaling components in six different cSCC cell lines, some of which can give rise to tumors when transplanted onto immunocompromised mice (see e.g. Boukamp et al., 1982; Tilgen et al., 1983). The analyses revealed a relatively heterogeneous expression pattern of HH signaling components in these tumor cell lines. *PTCH*, *GLI1*, and *GLI2* were expressed in all cell lines, but to a variable extend (**Figure 2**). Similarly, the expression level of *GLI3* and *SMO* varied from high expressers (MET-4) and non-expressers (SCC-13, SCL-2) (**Figure 2**). In contrast, only MET-1, MET-4 cells, and SCC-12 cells expressed the *SHH* ligand whereas SCL-1, SCL-2, and SCC-13 cells did not (**Figure 2A**).

Since these analyses revealed that some cSCC cell lines express all components necessary for stimulation of canonical HH signaling we next tried to modulate canonical HH signaling in MET-1, MET-4 and SCL-1 cells. The rationale for choosing these cell lines was their origin as well as their differential expression pattern of HH signaling components: The MET-1 cell line originated from a primary tumor that gave rise to a metastatic lesion from which the cell line MET-4 was generated (Popp et al., 2000). Both cell lines express high *GLI* and *SHH* levels (thus serving as a model for SHH positive cSCC described by Schneider et al., 2011). The SCL-1 cell line, which is derived from a poorly differentiated primary cSCC (Boukamp et al., 1982), expresses only moderate levels of HH signaling components but is *SHH* negative (serving as model for SHH negative cSCC).

However, the treatment of SCL-1, MET-1, and MET-4 cells with Shh-conditioned medium (Shh-CM) or the SMO agonist SAG for induction of canonical HH signaling did not result in upregulation of *GLI1* expression compared to the positive controls that verified the functionality of Shh-CM or SAG (**Figure 2B**). Similar results were obtained by analyzing GLI reporter activity in SCL-1 cells, in which incubation with Shh-CM did not change GLI-driven luciferase activity in comparison to the control (**Supplementary Figure S3A**). The cells, at least MET-4 cells, also do not possess primary cilia, which are essential for canonical HH signaling (**Supplementary Figure S3B**). Besides the unresponsiveness of the cell lines to HH signaling inducers the cells were also unresponsive to increasing concentrations of the SMO inhibitors cyclopamine (**Figure 2C**, upper panel) or vismodegib (**Figure 2C**, lower panel). In contrast, incubation of SCL-1 with HhA resulted in a paradoxical increase in *GLI1* expression, whereas this drug inhibited *GLI1* transcription in MET-1 and MET-4 cells (**Figure 2C**, middle panel).

Together, these results show that i) canonical HH signaling in cSCC cell lines is neither inducible with SHH or SAG nor inhibitable with cyclopamine or vismodegib, at least under our experimental conditions and ii) that the SMO inhibitor HhA can paradoxically induce the expression of *GLI1*, which has also been seen in other tumor cell lines (Ridzewski et al., 2015).

### Epidermal Growth Factor Decreases *GLI1* Expression in Cutaneous Squamous Cell Carcinoma Cell Lines

**Figure 3:**
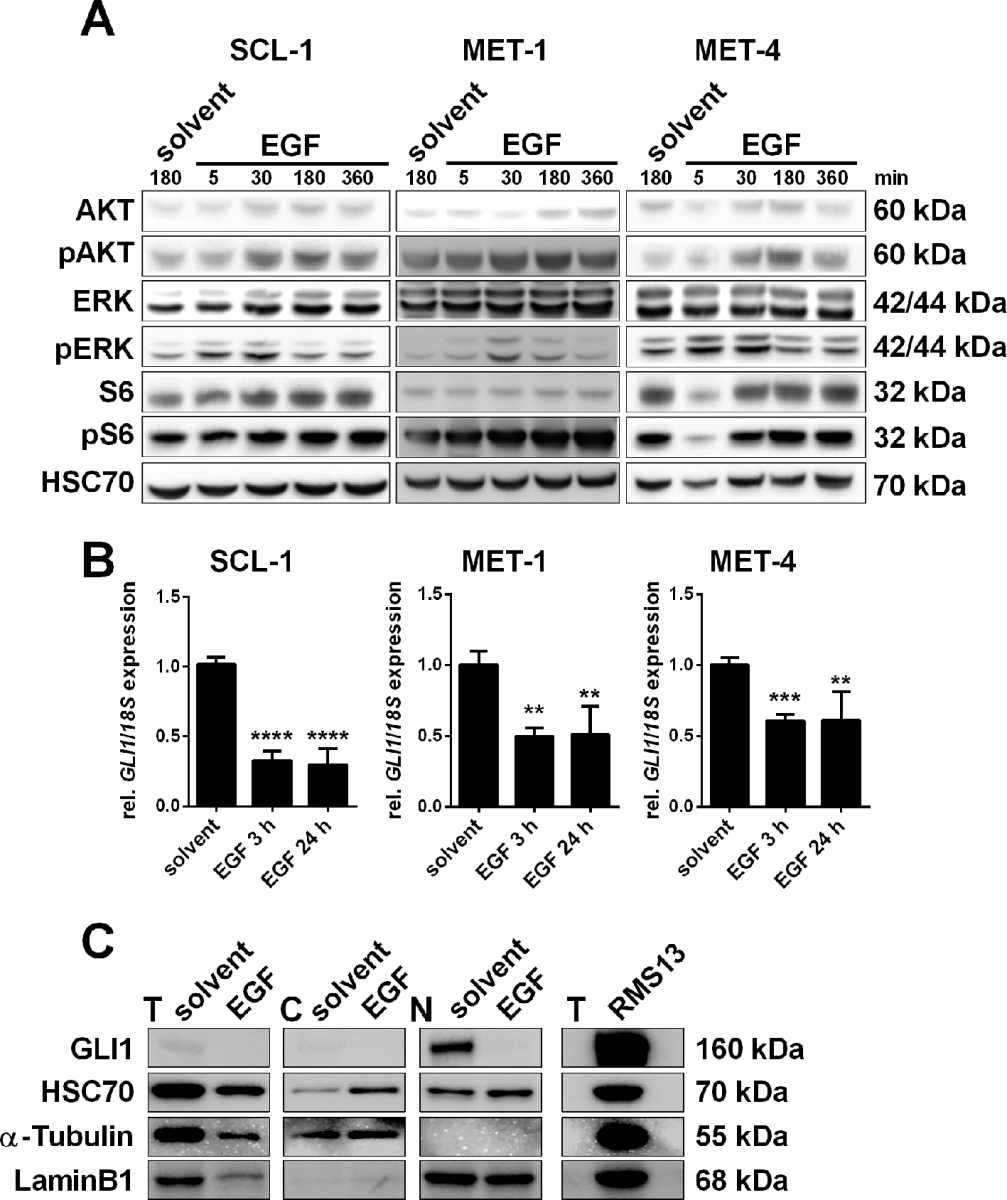
Impact of EGF treatment on human cSCC cell lines. **(A)** Representative Western blot analyses of SCL-1, MET-1, and MET-4 cells after treatment with 100 ng/µl EGF for 5, 30, 180, or 360 min or solvent for 180 min showing activation of PI3K/AKT, MEK/ERK, and mTOR signaling pathways upon EGF-treatment in a time-dependent manner. EGF already triggered phosphorylation of ERK after 5 min in SCL-1 and MET-4 cells, while phosphorylation of AKT and of S6 occurred after 30 min. Detection of HSC70 served as a loading control. **(B)** qRT-PCR-based *GLI1* expression analyses of SCL-1, MET-1, and MET-4 cells after treatment with 100 ng/µl EGF for 3 or 24 h. *GLI1* expression levels were normalized to *18S *rRNA gene expression and values of solvent treated controls were set to 1. Results represent mean values + SEM of two independent experiments measured in triplicates. Statistical significance was tested by a nonparametric Mann–Whitney test. **P < 0.01, ***P < 0.001, ****P < 0.0001. **(C)** Western blot analysis of total (T), cytosolic (C), and nuclear (N) fractions of MET-4 cells after treatment with 100 ng/µl EGF for 24 h. Despite unequal loading of the total protein fractions, the results clearly show that similar to reduced *GLI1* mRNA expression EGF also triggered downregulation of GLI1 protein. HSC70, α-Tubulin, or LaminB1 served as loading controls for total, cytosolic or nuclear lysates, respectively. Total protein lysate of RMS13 cells served as positive control for GLI1 protein expression. Protein sizes in kilodalton are indicated on the right side of the blots shown in **(A** and **C)**.

Despite the expression of HH signaling components a modulation of the canonical HH signaling *via* SMO activators or inhibitors is not possible in cSCC cells. Thus we next evaluated the impact of non-canonical HH signaling in cSCC cells and focused on other pathways that could be responsible for activation of GLI factors downstream of the HH/PTCH/SMO axis. Since the high expression of EGF and EGFR seems to play a very important role in cSCC (**Figure 1**; **Supplementary Figure S1E**) (Canueto et al., 2017), we hypothesized that EGFR signaling may regulate GLI activity *via* the RAS/MEK/ERK axis like in BCC (Schnidar et al., 2009; Eberl et al., 2012). To verify this hypothesis we incubated SCL-1, MET-1, and MET-4 cells that all express EGFR (Beier et al., 2006; Clayburgh et al., 2013) with recombinant EGF. Western blot analyses verified that an incubation with EGF as short as for 5 min already triggered the phosphorylation of ERK and thus activation of MEK/ERK signaling (**Figure 3A**). A phosphorylation of AKT and thus an activation of PI3K/AKT signaling appeared 30 min after EGF incubation (**Figure 3A**). While the EGF-induced levels of pAKT remained elevated until at least 360 min, phosphorylation of ERK decreased to basal levels after 180–360 min (**Figure 3A**). Simultaneously, EGF treatment significantly decreased *GLI1* transcription in all three cSCC cell lines up to 24 h (**Figure 3B**). Western blot analysis shows that this is accompanied by downregulation of GLI1 protein as well (**Figure 3C**). Together these data suggest that EGFR signaling negatively regulates *GLI1/*GLI1 expression in cSCC cell lines.

### Epidermal Growth Factor-Mediated *GLI1* Suppression in Cutaneous Squamous Cell Carcinoma Cell Lines Depends on MEK/ERK Signaling

**Figure 4:**
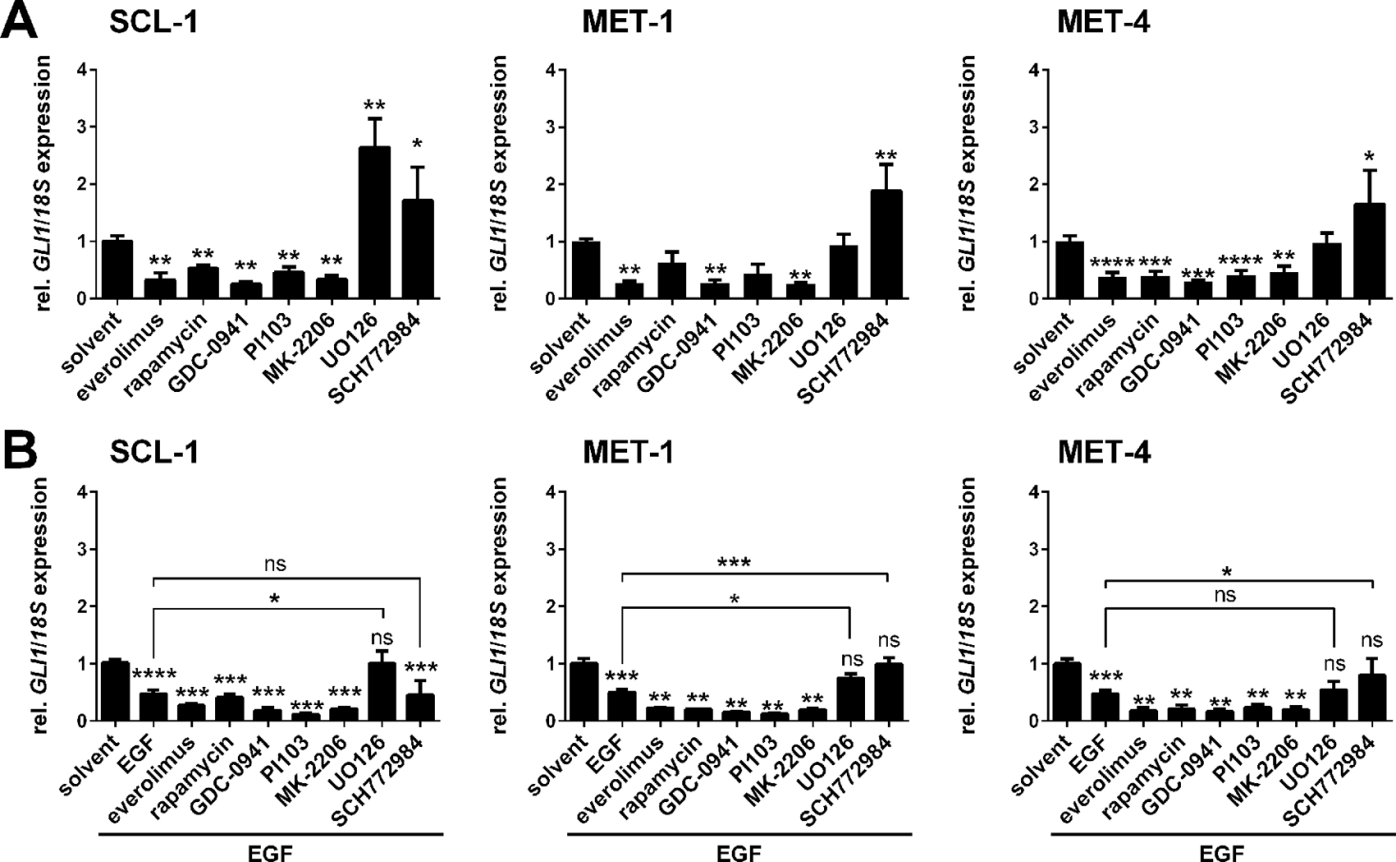
Effects of EGF and/or PI3K, AKT, mTOR, or MEK1/2 inhibition on *GLI1* expression level in human cSCC cell lines. qRT-PCR-based analysis of *GLI1* expression levels of SCL-1, MET-1 and MET-4 cells **(A)** after 24 h treatment with 50 nM everolimus, 100 nM rapamycin, 3 µM PI103, 10 µM GDC-0941, 5 µM MK-2206, 20 µM UO126, or 100 nM SCH772984 and **(B)** after 24 h treatment with the mentioned drugs and concomitant 3 h incubation with 100 ng/µl EGF. *GLI1* expression levels were normalized to *18S *rRNA gene expression and values of solvent treated controls were set to 1. Results represent mean values + SEM of three independent experiments measured in triplicates. Statistical significance was tested by a nonparametric Mann-Whitney test. *P < 0.05, **P < 0.01, ***P < 0.001, ****P < 0.0001; ns, not significant.

To analyze whether the EGF-induced *GLI1* inhibition was mediated by the downstream MEK/ERK or the PI3K/AKT/mTOR axes SCL-1, MET-1, and MET-4 cells were incubated with specific MEK/ERK (MEK inhibitor: UO126, ERK inhibitor: SCH772984) or PI3K/AKT/mTOR inhibitors (mTOR inhibitors: everolimus or rapamycin, specific PI3K inhibitor: GDC-0941, dual PI3K and mTOR inhibitor: PI103, specific AKT inhibitor: MK-2206) in presence or absence of EGF. After verification their efficiency by Western blot in all three cell lines (**Supplementary Figure S4**), *GLI1* expression levels were quantified. This approach revealed that treatment with PI3K/AKT and/or mTOR inhibitors consistently inhibited *GLI1* expression. In contrast, treatment with UO126 or SCH772984 significantly upregulated *GLI1* expression in SCL-1 cells or all cell lines, respectively (**Figure 4A**). Thus, *GLI1* transcription seems to be generally activated by PI3K/AKT/mTOR signaling but inhibited by the MEK/ERK axis.

Moreover the combination of EGF with PI3K/AKT and/or mTOR inhibitor treatment further strengthened the EGF-mediated downregulation of *GLI1* transcription by mTOR, PI3K, or AKT inhibitors (**Figure 4B**; please note that significances between single EGF and the combination treatments are not indicated in the figure). However, when EGF was combined with the MEK inhibitor UO126, *GLI1* levels were upregulated in comparison to EGF treatment alone and reached the basal *GLI1* level of the solvent treated control (**Figure 4B**). This tendency was observed in all three cell lines and was significant for SCL-1 and MET-1 cells (**Figure 4B**). Basal *GLI1* levels were also reached after combined treatment with EGF and the ERK inhibitor SCH772984 in MET-1 and MET-4 cells, but not in SCL-1 cells (**Figure 4B**). These data foster the assumption that the observed EGF-mediated *GLI1* downregulation is regulated by MEK (in SCL-1 cells) or by ERK (in MET-1 and MET-4 cells).

### Epidermal Growth Factor-Mediated *GLI1* Suppression Does Not Influence Proliferation of Cutaneous Squamous Cell Carcinoma Cell Lines

**Figure 5:**
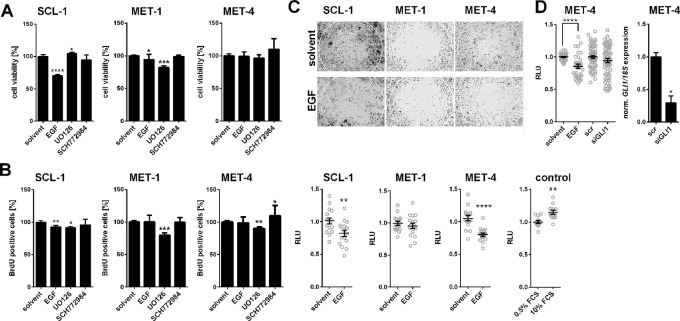
EGF-induced modulation of proliferation and migration of human cSCC cell lines. **(A)** WST-1 metabolic activity assay and **(B)** BrdU incorporation assay of SCL-1, MET-1, and MET-4 cells after 24 h treatment with 100 ng/µl EGF, 20 µM UO126, or 100 nM SCH772984. **(C)** Representative pictures (upper panel) and quantitative analyses (lower panel) of the migratory capacity of SCL-1, MET-1 and MET-4 cells treated with 100 ng/µl EGF measured by zone exclusion assays (see Material and Methods for detailed description). HT1080 cells cultured in 0.5 or 10% FCS for 12 h served as control for the cellular migration assay **(D)** Quantification of the migratory capacities of MET-4 cells after siRNA-mediated *GLI1* knock down (left panel) measured by zone exclusion assays. The siRNA-mediated *GLI1* knock down of MET-4 cells was verified by qRT-PCR (right panel). *GLI1* expression levels were normalized to *18S *rRNA gene expression. Results represent mean values + SEM of three independent experiments measured in triplicates **(A**,** B)** or of two independent experiments measured in triplicates [**(D)**, right panel], in octuplicates **(C)**, or in double octuplicates [**(D)**, left panel]. Measured values of the respective controls were set to 100% **(A**,** B)** or to 1 **(C**,** D)**. Statistical significance was tested by a nonparametric Mann–Whitney test. *P < 0.05, **P < 0.01, ***P < 0.001, ****P < 0.0001.

To analyze whether the EGF-mediated downregulation of *GLI1* alters metabolic activity or proliferation of cSCC cells, WST-1 and BrdU incorporation assays were performed on SCL-1, MET-1, and MET-4 cells after incubation with EGF or with UO126 or SCH772984 to block the EGF-mediated GLI1 suppression. As shown in **Figures 5A**, B EGF that generally suppressed *GLI1* transcription (see above), decreased the metabolic activity and proliferation of SCL-1 but not of MET-1 (with exception of a slight decrease in metabolic activity) and MET-4 cells. This was surprising since EGF is generally accepted as a growth factor (e.g. EGF induced proliferation of MCF-7 cells, which were used as published control cells (Garcia et al., 2006; data not shown). However, these results are in concordance with the literature describing that EGF can have toxic and antiproliferative effects on cSCC (Ponec et al., 1988; Commandeur et al., 2012). Furthermore, the results show that UO126, which increased *GLI1* expression solely in SCL-1 cells (see **Figure 4B**), slightly decreased proliferation of all cell lines, whereas SCH772984, which increased the *GLI1* level in all cell lines (see **Figure 4B**) was neither toxic nor antiproliferative for any of the cells (**Figures 5A, B**). These results emphasize that the *GLI1* expression level is not necessarily associated with the proliferative capacity of cSCC cells. Indeed, siRNA-mediated *GLI1* knock down (**Supplementary Figure S5A**) as well as *GLI1* overexpression (**Supplementary Figure S5B**) also did not affect the proliferation rate of MET-4 cells. Identical results were obtained when murine *Gli1* was overexpressed (data not shown). Together these data indicate that the *GLI1 *expression level is not related to cellular proliferation in cSCC cell lines.

### Epidermal Growth Factor Inhibits Cutaneous Squamous Cell Carcinoma Cell Migration in a GLI1-Independent Manner

EGFR signaling has been implicated in stimulation of cSCC migration (e.g. Zhang et al., 2018). Therefore, we next analyzed whether EGF-mediated *GLI1* suppression plays a role in modulation of tumor cell motility. For this purpose we monitored the effect of EGF by a zone exclusion assay. As shown in **Figure 5C** incubation of the cells with EGF significantly decreased the migratory capacity of SCL-1 and MET-4 cells, whereas this effect was not seen in MET-1 cells (**Figure 5C**). In order to test whether GLI1 is involved in this process, we also knocked-down *GLI1* by siRNA technology in MET-4 cells (see **Figure 5D**, right panel). However, the *GLI1* knockdown did not influence migration of the cells, at least not in the used experimental setting (**Figure 5D**). Together these data show that EGF can inhibit migration of least a subgroup of cSCC cells and that GLI1 apparently does not play a role in this process.

### Association of Lower *GLI1* Level in the Skin With Promotion of Chemically-Induced Papilloma Formation

**Figure 6:**
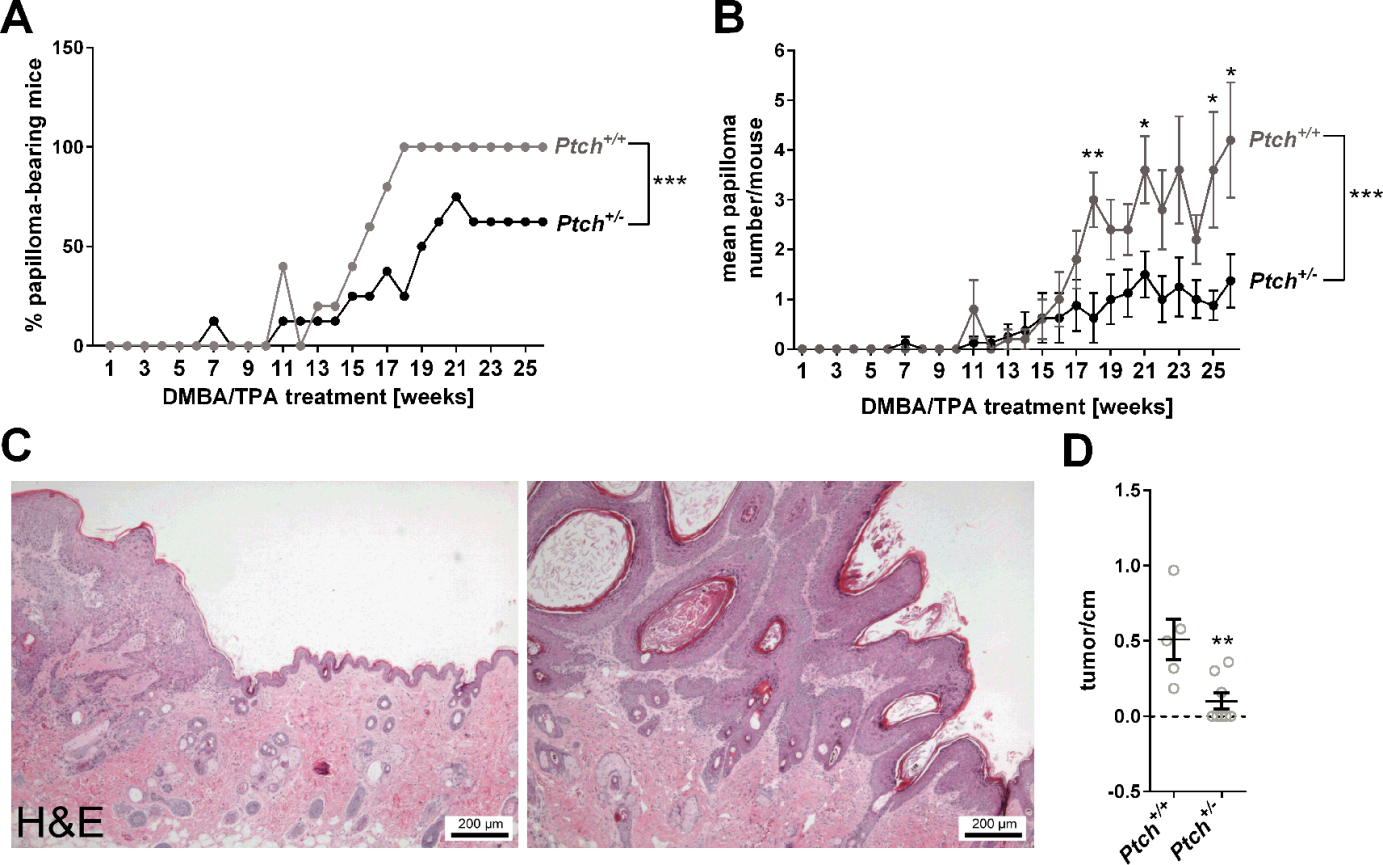
DMBA/TPA-induced papilloma development in *Ptch* *^+/+^* and *Ptch* *^+/-^* mice. **(A)** Percentage of papilloma-bearing mice and **(B)** mean papilloma number per mouse ( ± SEM) after initial single DMBA- and following 26 weeks TPA treatment of heterozygous *Ptch* mutant (*Ptch* *^+/-^*, n = 8) and *Ptch* wildtype mice (*Ptch* *^+/+^*, n = 5). **(C)** Hematoxylin/eosin-stained representative DMBA/TPA-induced papilloma with keratoacanthoma-like features and **(D)** numbers of these tumors per cm back skin of *Ptch* *^+/+^* and *Ptch* *^+/-^* mice. Statistical significance was tested by a nonparametric Wilcoxon matched-pairs signed rank test **(A**,** B)** or a nonparametric Mann–Whitney test **(B**,** D)**. *P < 0.05, **P < 0.01, ***P < 0.001.

Recently, it has been shown that the skin of heterozygous *Ptch* *^+/-^* mice express higher levels of *Gli1* when compared to skin derived from *Ptch* *^+/+^* wildtype mice (Svard et al., 2008). In order to analyze whether the *Gli1* expression level of the skin is associated with papilloma formation, *Ptch* *^+/+^* and *Ptch* *^+/-^* mice that indeed express *Gli1* at different level (**Supplementary Figure S6**) were subjected to the two stage DMBA/TPA carcinogenesis protocol. Interestingly, only 62.5% *Ptch* *^+/-^* mice but 100% of the *Ptch* *^+/+^* control mice developed macroscopically visible skin tumors 26 weeks after the initial DMBA treatment (**Figure 6A**). Moreover, the latency time as well as the tumor load per animals was significantly lower in *Ptch* ^+/-^ mice [tumor-bearing mice after 18 weeks: 25% (3/8 mice); tumor load/mouse after 26 weeks: 1.373 +/- 0.532] compared to the controls [tumor-bearing mice after 18 weeks: 100% (5/5 mice); tumor load/mouse after 26 weeks: 4.2 +/- 1.158] (**Figures 6A, B**). Microscopical examination of the skin after the 26-weeks treatment period revealed that both *Ptch* *^+/+^* and *Ptch* *^+/-^* mice have developed papilloma with keratoacanthome-like features (**Figure 6C**). However, the number of these tumors per cm skin was significantly lower in *Ptch* *^+/-^* mice compared to *Ptch* *^+/+^* controls (**Figure 6D**).

Together these data indicate that skin of *Ptch* *^+/-^* mice, which express higher *Gli1* levels compared to *Ptch* *^+/+^* skin is less susceptible to chemically-induced papilloma formation than wildtype skin. In other words, lower *Gli1* level in the skin is associated with promotion of chemically-induced cSCC formation.

## Discussion

The importance of canonical HH signaling is well known for BCC. However, the relevance of canonical HH signaling pathway for cSCC is far from clear. On the one hand, canonical HH signaling has been proposed to promote proliferation, migration and invasiveness of cSCC (Sun et al., 2016). On the other hand numerous clinical data demonstrate that inhibition of canonical HH signaling i.e. BCC treatment with vismodegib can result in cSCC development (e.g. Mohan et al., 2016).

In contrast to Schneider and colleagues, who described immunohistochemical staining for SHH in 67% and for GLI1 in 42% of human cSCC samples (Schneider et al., 2011) our data revealed that none of the 10 cSCC analyzed express SHH. However, distinct tumor nests of approximately 50% of the tumors express *GLI1*, which is the major downstream target of HH signaling. Currently it is not known what drives *GLI1* transcription in these tumor cells. However, although we were not able to reliably analyze the expression of the two other HH ligands (i.e. DHH or IHH), the circumstance that it was impossible to induce or inhibit *GLI1* expression in cSCC cell lines with specific HH modulators do not support canonical HH signaling in cSCC, at least not in our setting. In fact, our data rather supports non-canonical modulation of HH signaling in the tumors. Thus, our experiments using PI3K, AKT and mTOR inhibitors implicate that *GLI1* transcription in cSCC is activated by the PI3K/AKT/mTOR axis. Nevertheless, these data should be handled with caution because WST-based assays revealed strong toxicity of the inhibitors (data not shown), which could have resulted in unreliable *GLI1* expression data. This is different for EGF and the used MEK and ERK inhibitors. Although EGF was toxic to one of the cSCC cell lines, our data show that *GLI1* transcription is unambiguously suppressed by the EGF/MEK/ERK axis (see **Figures 3B** and **4B**). This negative regulation of HH signaling by MEK/ERK is similar to medulloblastoma, in which FGF-mediated ERK activation inhibits HH pathway target gene expression including *GLI1* (Fogarty et al., 2007b; Emmenegger et al., 2013). It is also comparable to squamous cell carcinoma of the head and neck, in which EGFR signaling downregulates *GLI1* (Keysar et al., 2013). Finally, this observation fits to the ubiquitous expression of EGF in human cSCC samples and the strong expression of EGFR (see **Figure 1** and **Supplementary Figure S1E**). Unfortunately, and despite the fact that we used adjacent tumor sections, we are currently not able to judge whether *GLI1* positive tumor cells are completely devoid of EGFR expression. However, the fact that EGFR signaling suppresses the expression of the major HH target *GLI1* in cultured cSCC cells opens the intriguing possibility that the same mechanism is responsible for *GLI1* suppression in the *in vivo* situation.

Our *in vitro* experiments also suggest that neither EGFR signaling nor GLI1 are necessary for cSCC proliferation. This is at the first glance surprising because human cSCC tumors highly express both EGFR and the proliferation marker Ki67 at the tumor invasion front (see **Figure 1**) and EGFR signaling has been implicated in cSCC proliferation (El-Abaseri et al., 2005; Canueto et al., 2017). In addition, there are also two reports showing that the EGFR inhibitor cetuximab inhibits proliferation of cSCC cell lines (Galer et al., 2011; Clayburgh et al., 2013). However, the experiments were done by MTT assay, which rather mirrors cellular viability and not necessarily the proliferation rate. Therefore, it is possible that cetuximab is just toxic to cSCC cells. This also could explain the results of currently ongoing cetuximab clinical trials, which show that the response duration to cetuximab monotherapy is short (Dereure et al., 2016) or missing (Berliner et al., 2019).

Curiously, our *in vitro* data also show that EGFR signaling apparently counteracts cSCC migration. This has been demonstrated by zone exclusion assays in SCL-1 and MET-4 cell lines. The circumstance that this effect was not seen in MET-1 cells may indicate specificity for only a subset of cSCC. At the first glance *inhibition* of cellular migration upon EGF treatment seems paradoxical, because EGFR signaling has been implicated in *stimulation* of cSCC migration (e.g. see Zhang et al., 2018). Since we used a rather high concentration of EGF that apparently was slightly toxic to SCL-1 and MET-1 cells, it is tempting to speculate that cellular migration was hampered by toxicity. However, this is unlikely since EGF also inhibited migration of MET-4 cells without exerting any toxic effects. It is more likely that cultured cSCC cells lack clathrin-mediated polarized endocytosis of EGF bound to EGFR, which is required for EGF-directed migration (Belleudi et al., 2011; Mutch et al., 2014). Yet this is pure speculation and remains to be analyzed in the future. Finally, our experiments suggest that inhibition of migration does not involve GLI1, because a respective *GLI1* knock down does not affect the migratory capacity of at least MET-4 cells. This is different for many other tumors e.g. for breast and pancreatic cancer (Inaguma et al., 2011; Kwon et al., 2011), in which HH/GLI1 signaling is connected to migration and invasiveness. Nevertheless, although the lack of GLI1-mediated consequences could be simply a characteristic of the used cSCC cell lines, our data strongly suggest that GLI1 does not play an important role in growth, progression or aggressiveness of already established cSCC. This is revealed by infrequent and weak expression of *GLI1* in single tumor areas in 50% of cSCC and by the absence of cSCC growth changes upon *GLI1* downregulation.

In contrast, low levels of *GLI1* expression seem to be associated with cSCC initiation. This is due to the fact that murine skin expressing normal *Gli1* level is significantly more susceptible to chemically-induced papilloma formation than skin with slightly elevated *Gli1* levels. This observation was made in *Ptch* *^+/+^* or *Ptch* *^+/-^* skin, respectively. Interestingly, experiments by Wakabayashi and colleagues revealed similar results. Their data in K5Hras mice suggest that Ptch is needed for cSCC initiation but not for later cSCC stages. This led to the hypothesis that the presence of Ptch and inactive Hh/Gli signaling is a prerequisite for cSCC development, whereas loss of Ptch and thus activation of Hh/Gli signaling is a prerequisite for BCC (Wakabayashi et al., 2007). Indeed, BCC and cSCC can have the same cellular origin including stem cells of the hair follicle (see Thieu et al., 2013; Peterson et al., 2015 for review).

The “GLI1” level hypothesis in cSCC and BCC development together with our observation that low levels of *GLI1* expression seem to be associated with cSCC initiation could also explain the observation that treatment of BCC with vismodegib, which usually inhibits canonical HH signaling pathway by binding to SMO, can result in cSCC development (see e.g. Ransohoff et al., 2015; Mohan et al., 2016). Thus, similar what has been discussed by other labs (i.e. Ransohoff et al., 2015), the transition from a BCC in a cSCC could be due to a vismodegib-induced downregulation of GLI1 in the BCC. Since cellular origins of BCC and cSCC can be the same (see above), GLI1 downregulation may result in the induction of an alternative (tumor) stem cell fate decision, which—if the stem cell has acquired mutations in cSCC-driving oncogenes or tumor suppressor genes such as RAS, TP53, NOTCH1/2, or MLL3 (Pickering et al., 2014)—results in the appearance of a squamous phenotype.

If all these assumptions turn out to be true, one could propose a new model for cSCC formation and maintenance, in which EGF (that is expressed throughout the cSCC-bearing skin) signals *via* EGFR and the MEK/ERK axis to repress *GLI1* transcription with the latter step being essential for cSCC pathogenesis. However, this is pure speculation and remains to be validated in the future.

## Data Availability Statement

All datasets generated for this study are included in the article/ **Supplementary Material**.

## Ethics Statement

Ten biopsy specimens from human invasive cSCC were studied. Histopathology of all cases was centrally reviewed. Materials were obtained at a time when patients gave their general consent to use of their materials for scientific purposes, so additional informed consent for this particular study was required. The respective approval by our local ethics committee has been obtained for staining of tumor markers in epithelial skin tumors (file number 19/3/02). DMBA/TPA treatments of mice were performed in compliance with all German legal and ethical requirements (file number 33.9-42502-04-100/07).

## Author Contributions

JP, NK, AU, and HH contributed to conception and design of the study. JP, NK, MK, JB, SZ, FN, PB, H-US, and AU contributed to data acquisition and helped with data interpretation. SE, MS, and PB provided reagents and ideas. AU and HH wrote the manuscript. All authors read and approved the manuscript.

## Funding

This work was supported by the grant 2013.058.1 from the Wilhelm Sander Foundation to HH, SE, and MS.

## Conflict of Interest

The authors declare that the research was conducted in the absence of any commercial or financial relationships that could be construed as a potential conflict of interest.
