# Supplementary material for: Regulation and Role of GLI1 in Cutaneous Squamous Cell Carcinoma Pathogenesis
Source: Front Genet. 2019 Dec 4;10:1185. doi: 10.3389/fgene.2019.01185 (PMC6904360; doi:10.3389/fgene.2019.01185)
Supplement: Supplementary file 1 [file DataSheet_1.pdf]

Supplementary Material

Regulation and role of GLI1 in cSCC pathogenesis

Joanna Pyczek, Natalia Khizanishvili, Maria Kuzyakova, Sebastian Zabel, Julia Bauer, Frauke Nitzki, Steffen Emmert, Michael P. Schön, Petra Boukamp, Hans-Ulrich Schildhaus, Anja Uhmann, Heidi Hahn*

*** Correspondence:** Heidi Hahn; hhahn@gwdg.de

1 Supplementary Figures and Tables

1.1 Supplementary Tables

Supplementary Table S1: Primary and secondary antibodies used for immunohistochemical, immunofluorescent and Western Blot analyses.

**primary antibodies for IHC**	**dilution**	**antigen retrieval**
mAb rabbit anti-GLI1 (C68H3)<br>Cell signaling, 3538	1:200	citric acid pH 6
mAb mouse anti-EGFR<br>Dako M7239 (clone 30)	1:50	protease K
pAb rabbit anti-pS6 (Ser240/244)<br>Cell signaling	1:200	citric acid pH 6
pAb rabbit anti-SHH<br>Abcam, ab73958	1:100	citric acid pH 6
mAb mouse anti-DHH<br>Santa Cruz H-12 (sc-271101)	1:100/1:400	citric acid pH 6<br>Tris/EDTA pH9
pAb mouse anti-DHH<br>Novus Biologicals (#H00050846-B01P)	1:100/1:400	citric acid pH 6<br>Tris/EDTA pH9
mAb mouse anti-IHH<br>Santa Cruz F-9 (sc-271168)	1:100/1:400	citric acid pH 6<br>Tris/EDTA pH9
pAb mouse anti-EGF<br>R&D Systems	1:40	boric acid
pAb rabbit anti-SOX9<br>Merck, AB5535	1:2500	citric acid pH 6
mAb mouse anti-Ki67<br>BD Pharmingen 556003	1:50	citric acid pH 6
**secondary antibodies for IHC**	**dilution**	
En vision+ anti-rabbit/mouse/HRP*<br>Dako K5007	1:1 in TBS	
**primary antibodies for immunofluorescent stainings**	**dilution**	
acetylated tubulin mouse mAb<br>Sigma-Aldrich (clone 6-11B-1)	1:100	

**secondary antibodies for immunofluorescent stainings**	**dilution**
donkey anti-mouse Alexa488<br>Jackson ImmunoResearch (#715-545-150)	1:200
**primary antibodies for Western Blot**	**dilution**
pAb rabbit anti-GLI1 (V812)<br>Cell signaling, 2534	1:750
mAb mouse anti-α-Tubulin<br>Dianova, DLN-009992 (clone DM1A)	1:1000
pAb rabbit anti- Lamin B1<br>Cell signaling, 9087	1:1000
mAb mouse anti-AKT<br>BD Bioscience, 610861	1:1000
mAb rabbit anti-pAKT (Ser473)<br>Cell Signaling, 193H12	1:1000
pAb rabbit anti-MAP Kinase (ERK1, ERK2)<br>Sigma Aldrich Chemistry GmbH	1:1000
pAb rabbit anti-p44/42 MAPK (Erk1/2)<br>New England Biolabs GmbH	1:1000
mAb mouse anti-S6<br>Cell Signaling, 54D2	1:1000
pAb rabbit anti-pS6 (Ser240/244)<br>Cell Signaling	1:1000
mAb mouse anti-HSC70<br>Santa Cruz, sc-7298	1:5000
**secondary antibodies for Western Blot**	**dilution**
pAb sheep anti-mouse/HRP**<br>GE Healthcare, NA931	1:10000
pAb goat anti-rabbit/HRP**<br>Sigma-Aldrich, A0545	1:10000

* antibody binding was visualized using DAB+ (En vision+ system-HRP, Dako) or aminoethylcarbazol as chromogen.
** signals were visualized using the ECL plus detection system (GE Healthcare).
abbreviations: HRP, horseradish peroxidase; IHC, immunohistochemistry; mAb, monoclonal antibody; pAb, polyclonal antibody

Supplementary Table S2: Oligonucleotides used for qRT-PCR

Primer name	Primer sequence (5' - 3' orientation)	Location	Application
18S_For	CGCAAATTACCCACTCCCG	exon 1	*18S* expression
18S_Rev	TTCCAATTACAGGGCCTCGAA	exon 1	
GLI1 _For	AGCTACATCAACTCCGGCCA	exon 11	*GLI1* expression
GLI1 _Rev	GCTGCGGCGTTCAAGAGA	exon 12	
GLI2_For	AAGCCCTTCAAGGCGCAGTA	exon 9	*GLI2* expression
GLI2_Rev	TCGTGCTCACACACATATGGCTT	exon 10	
GLI3_For	GCCAGCGCAGCCCCTAT	exon 6	*GLI3* expression
GLI3_Rev	CGGCCTGGCTGACAGCCT	exon 7	
SMO_For	CAAGAACTACCGATACCGTGC	exon 6	*SMO* expression
SMO_Rev	AGCATGGTCTCGTTGATCTTGC	exon 7	
SHH_For	CAGCGACTTCCTCACTTTCC	exon 3	*SHH* expression
SHH_Rev	GGAGCGGTTAGGGCTACTCT	exon 3	
PTCH_For	GAGGTTGGTCATGGTTACATGGA	exon 6	*PTCH* expression
PTCH_Rev	TGCTGTTCTTGACTGTGCCACC	exon 7	

1.2 Supplementary Figures

Supplementary Figure S1

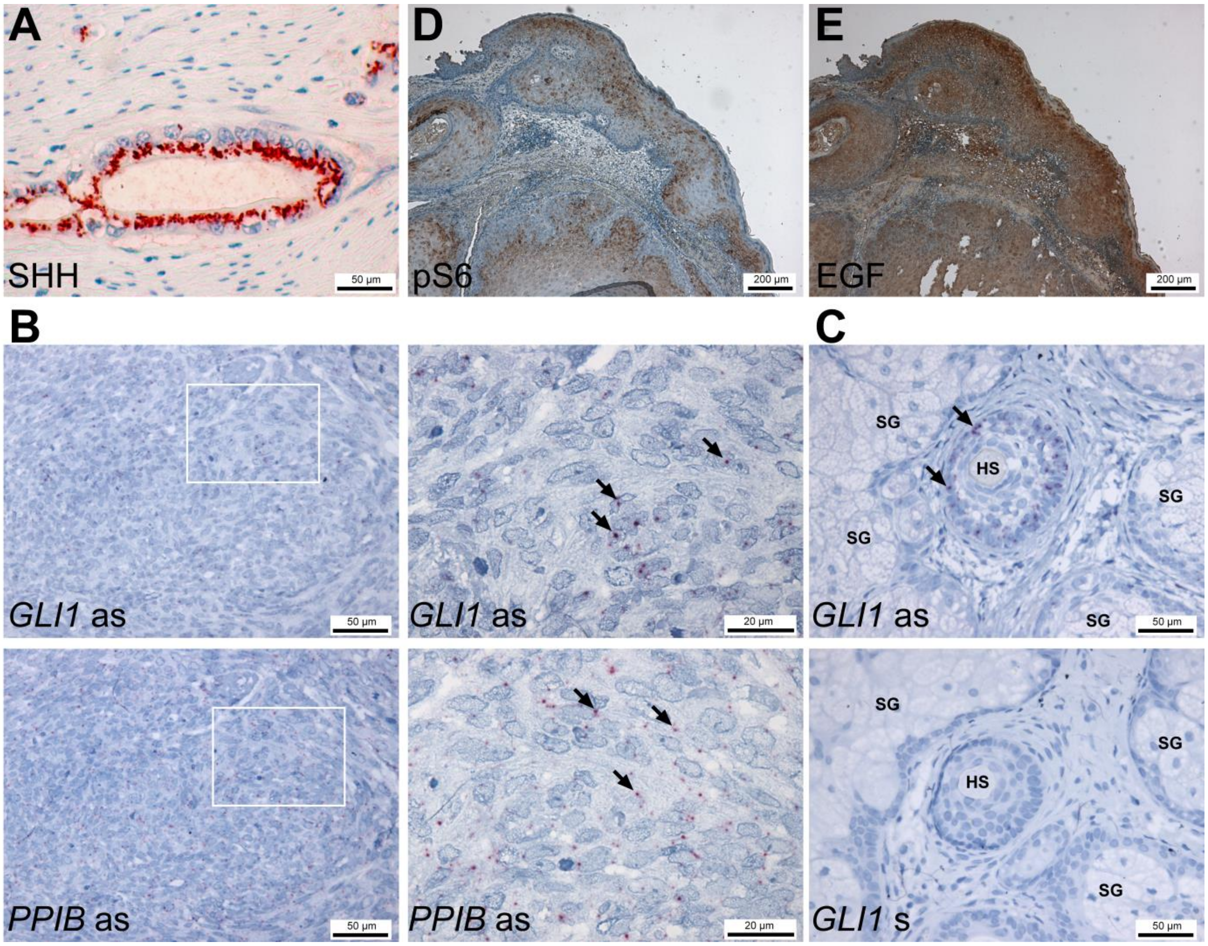

Supplementary Figure S1: (A) Section of a human pancreatic adenocarcinoma sample stained with the polyclonal anti-SHH antibody from Abcam (ab73958). The malignant epithelium of the adenocarcinoma samples shows strong positivity and thus resembles the described SHH expression pattern (Thayer et al. 2003). **(B)** Human BCC sample stained with antisense *GLI1* (*GLI1* as) and *PPIB* (*PPIB* as) RNA-probes by RNAscope® *in situ* hybridization. White boxes in the left panel indicate the magnification area shown in the right panel. **(C)** Representative section of a hair follicle within a section of a human cSCC biopsy stained with antisense (*GLI1* s) and sense *GLI1* (*GLI1* as) RNA-probes by RNAscope® *in situ* hybridization. SG, sebaceous gland; HS, hair shaft. Arrows in (B) and (C) indicate *GLI1*$^{+}$ or *PPIB*$^{+}$ cells. **(D)** Representative anti-pS6 and (E) anti-EGF antibody stainings of a human cSCC biopsy using the polyclonal anti-pS6 antibody from cell signaling or the polyclonal anti-EGF antibody from R&D Systems, respectively.

Supplementary Figure S2

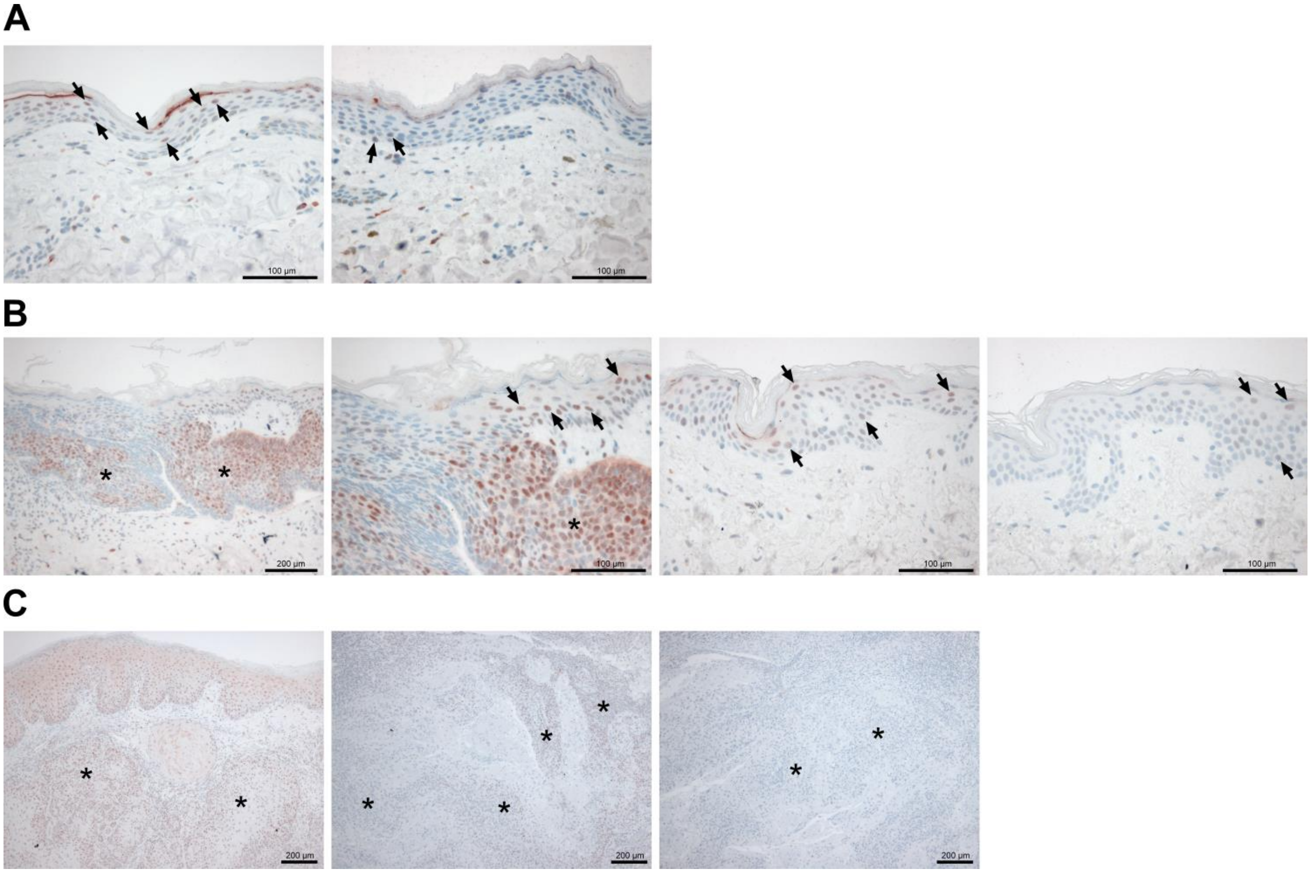

Supplementary Figure S2: Immunohistochemical analysis of skin tissue samples using the monoclonal anti-GLI1 C68H3 antibody from Cell signaling. (A) In normal human skin areas of the epidermis, in which nuclei of all layers are GLI1 positive were detected (Fig. S2A, left panel). In other areas GLI1 positive nuclei are detected in the basal layer (Fig. S2A, right panel). However, to our knowledge GLI1 has never been traced to the suprabasal layers. Normally, it is expressed in the stem cell compartment of the hair follicle (Abe et al. 2017) and also can be found in the stem cell compartment/basal layer of the interfollicular epidermis (Brownell et al. 2011) (as also shown in Fig. S2A; right panel). **(B)** Section of BCC shows strong GLI1-positivity (Fig. S2B; first picture from left) and the overlaying epidermis is partly positive for GLI1 (Fig. S2B; second picture from left). This is in accordance with data published by Dahmane et al (Dahmane et al. 1997). However, the adjacent normal epidermis again shows a heterogeneous GLI1 pattern with GLI1 positive cells (Fig. S2B; third picture from left) or rather GLI1 negative cells (Fig. S2B; forth picture from left) in suprabasal layers. The positivity of GLI1 in the BCC-adjacent normal interfollicular epidermis is in contrast to data described by Dahmane et al (Dahmane et al. 1997). **(C)** Sections of cSCC either stain completely positive or negative for GLI1 (Fig. S2C, left and right panels, respectively), whereas some cSCC contain both GLI1-positive and GLI1-negative tumor areas (Fig. S2C, middle panel). Asterisks indicate tumor areas; arrows point to GLI1 positive nuclei.

Supplementary Figure S3

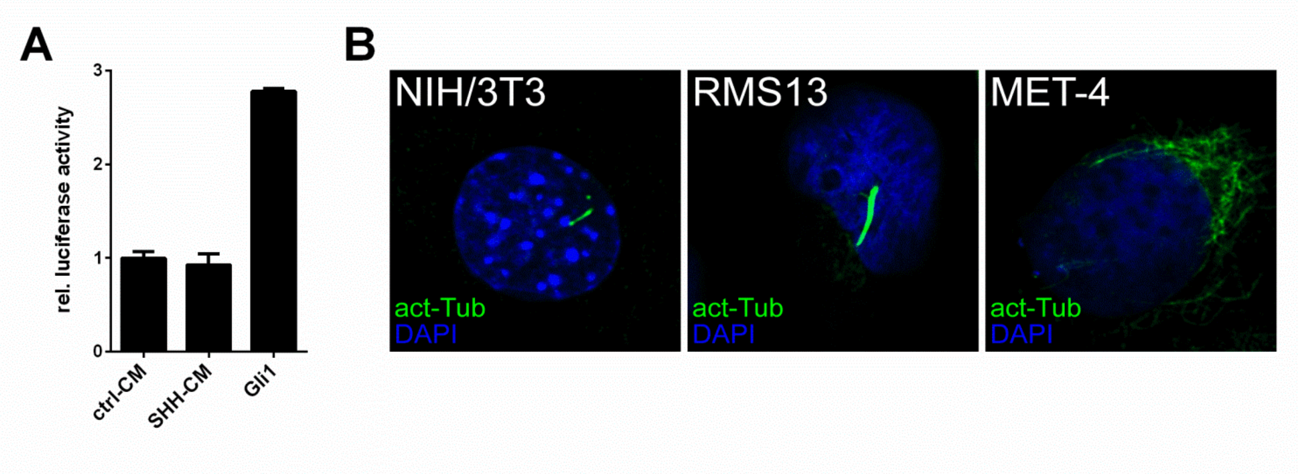

Supplementary Figure S3: (A) Relative luciferase activity of SCL-1 cells transfected with a plasmid encoding the firefly luciferase under the HSV TK promoter containing 9xGli-BS and incubated with either control- (ctrl-CM) or Shh-conditioned medium (Shh-CM). Co-transfection of a *Gli1* encoding plasmid served as a positive control (Gli1). The graph represents mean values + SEM of two experiments performed in three technical replicates. Values of control-CM treated cells were set to 1. **(B)** Visualization of primary cilia by immunofluorescent anti-acetylated tubulin (act-Tub) antibody stainings of NIH/3T3, RMS13 (positive controls) and MET-4 cells. DAPI reagent was used for counterstaining of the nuclei. Stainings were documented at 4800-fold magnification on a confocal laser scanning microscope equipped with software Fluoview FV100 (Olympus Corporation).

Supplementary Figure S4

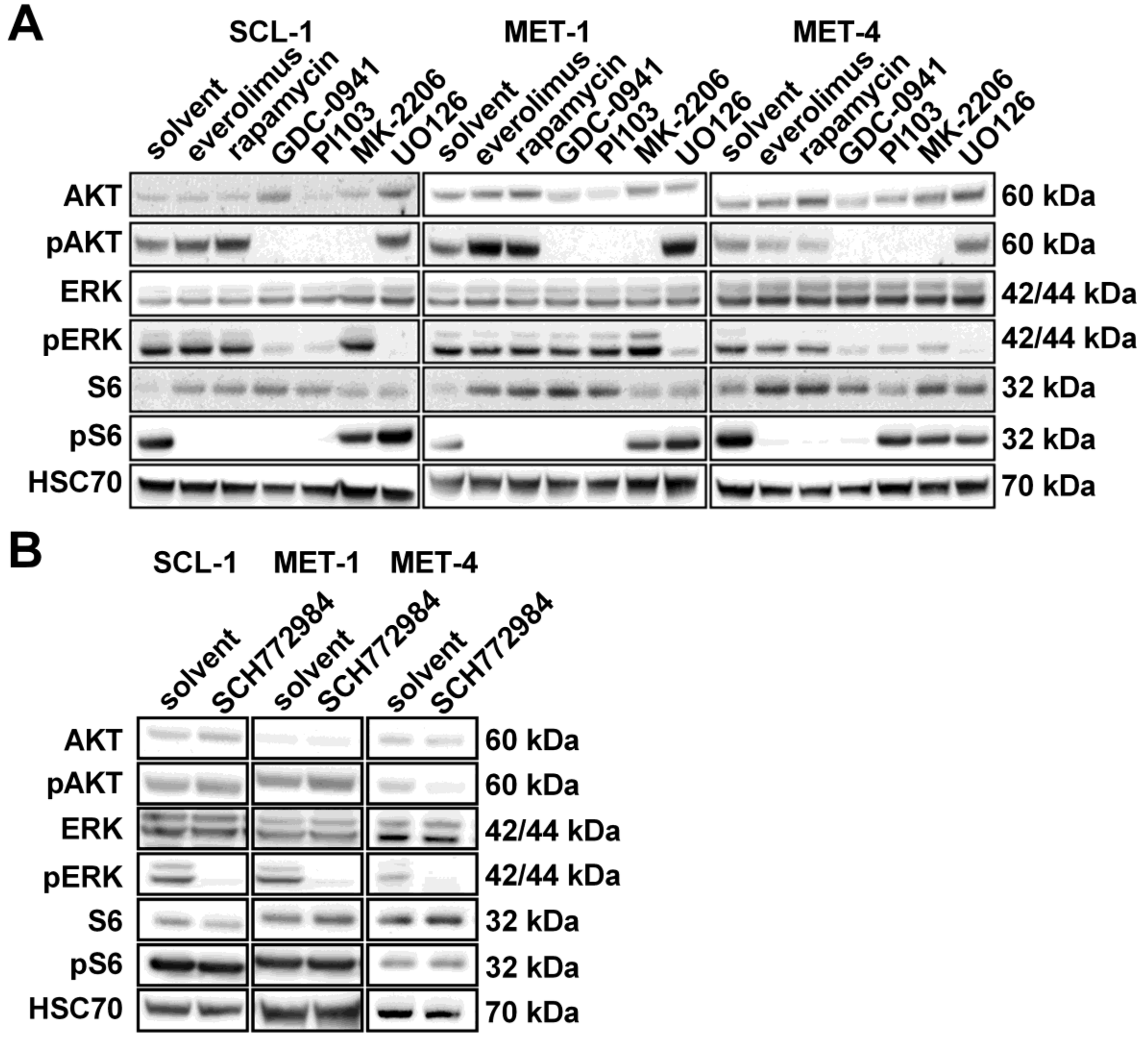

Supplementary Figure S4: Representative Western blot analyses of SCL-1, MET-1 and MET-4 cells after treatment with **(A)** solvent, 50 nM everolimus, 100 nM rapamycin, 3 µM PI103, 10 µM GDC-0941, 5 µM MK-2206 or 20 µM UO126 and **(B)** solvent or 100 nM SCH772984 for 24 h. The drugs efficiently inhibited the respective kinases in all 3 cell lines. Thus, everolimus and rapamycin reduced phosphorylation of the ribosomal protein S6 due to inhibition of mTORC1 and thus S6K. PI103 as a dual inhibitor of PI3K and mTOR decreased levels of pAKT and pS6. GDC-0941 and MK-2206 that block PI3K and AKT activity, respectively, reduced AKT phosphorylation level, whereas UO126 and SCH772984 efficiently reduced pERK levels. Detection of HSC70 served as a loading control. Protein sizes in kDA are indicated on the right side of the blots.

Supplementary Figure S5

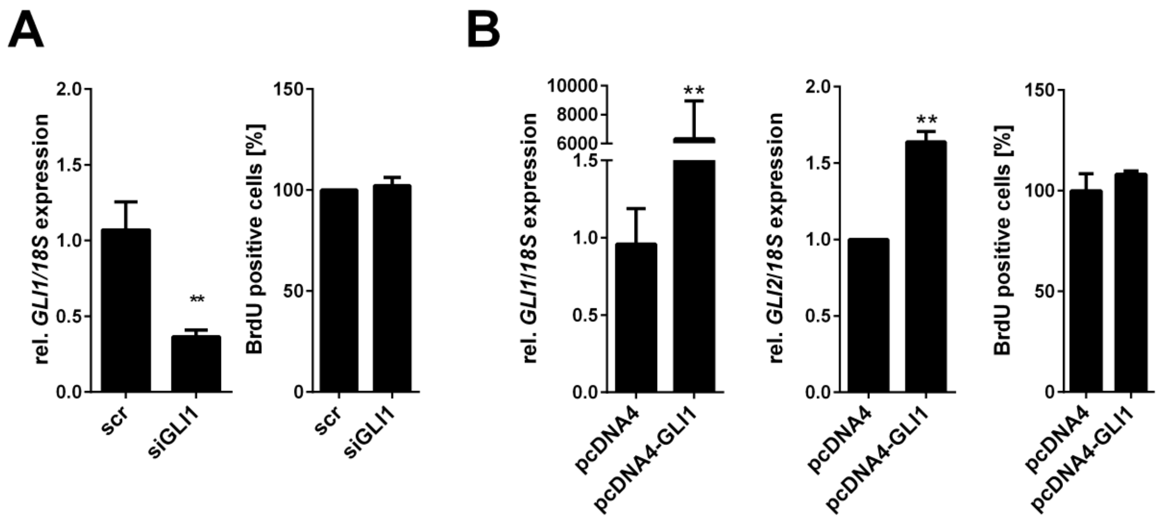

Supplementary Figure S5: (A) siRNA-mediated *GLI1* knock down and **(B)** GLI1 overexpression in MET-4 cells after 24 h were analyzed by qRT-PCR-based *GLI1* (**(A, B)**, left panels) and *GLI2* (**(B)**, middle panel) mRNA quantification and by BrdU incorporation measurements (**(A, B**), right panels). Gene expression levels were normalized to *18S rRNA* gene expression. Values of scrambled siRNA (**(A)**, scr) or empty vector (**(B)**, pcDNA4) transfected cells were set to 1 (qRT-PCR) or 100% (BrdU incorporation). Results represent mean values + SEM of two independent experiments measured in triplicates. Statistical significance was tested by a nonparametric Mann-Whitney test. ***P*<0.01.

Supplementary Figure S6

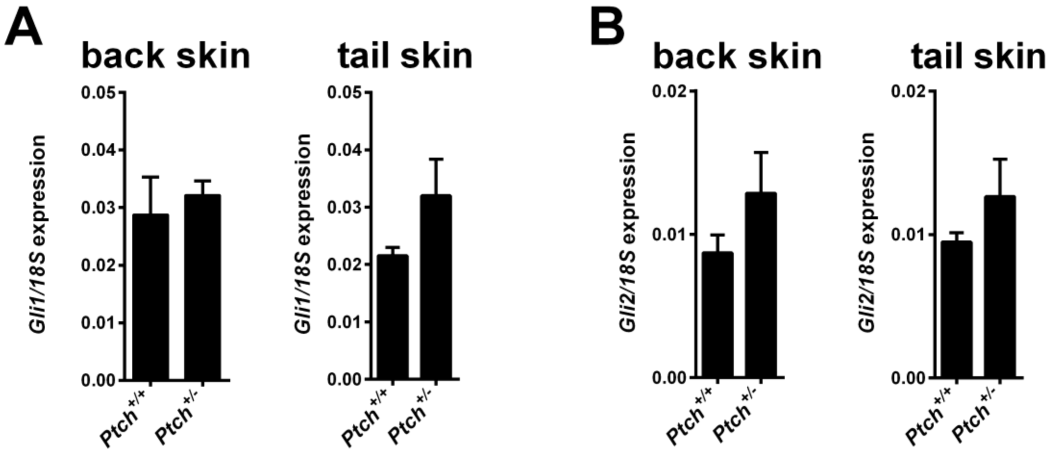

Supplementary Figure S6: qRT-PCR-based analysis of **(A)** *Gli1* and **(B)** *Gli2* expression levels in back (left panels) and tail skin (right panels) of 8-weeks old heterozygous *Ptch* mutant (*Ptch*$^{+/-}$, n=3) and wildtype *Ptch* (*Ptch*$^{+/+}$; n=3) mice. *GLI1* expression levels were normalized to *18S rRNA* gene expression. Results represent mean values + SEM of three mice per genotype measured in triplicates.

References

Thayer, S.P., di Magliano, M.P., Heiser, P.W., Nielsen, C.M., Roberts, D.J., Lauwers, G.Y., et al. (2003). Hedgehog is an early and late mediator of pancreatic cancer tumorigenesis. *Nature* 425, 851-6. doi:10.1038/nature02009.

Abe, Y., and Tanaka, N. (2017). Roles of the Hedgehog Signaling Pathway in Epidermal and Hair Follicle Development, Homeostasis, and Cancer. *J Dev Biol* 5, doi:10.3390/jdb5040012.

Brownell, I., Guevara, E., Bai, C.B., Loomis, C.A., and Joyner, A.L. (2011). Nerve-Derived Sonic Hedgehog Defines a Niche for Hair Follicle Stem Cells Capable of Becoming Epidermal Stem Cells. *Cell Stem Cell* 8, 552-565. doi:10.1016/j.stem.2011.02.021.

Dahmane, N., Lee, J., Robins, P., Heller, P., and Ruiz i Altaba, A. (1997). Activation of the transcription factor Gli1 and the Sonic hedgehog signalling pathway in skin tumours. *Nature* 389, 876-81. doi:10.1038/39918.
